# Role of Multimodal Imaging in Clinical Practice for the Diagnosis of Infective Endocarditis: A Case Series

**DOI:** 10.3390/idr16060099

**Published:** 2024-12-17

**Authors:** Sara Tordi, Giacomo Gonnelli, Maria Carolina Benvenuto, Daniele Rosignoli, Lisa Malincarne, Daniela Francisci

**Affiliations:** Infectious Diseases Clinic, Santa Maria della Misericordia Hospital, Department of Medicine and Surgery, University of Perugia, 06132 Perugia, Italy; giacomo.gonnelli@ospedale.perugia.it (G.G.); mcarolina.benvenuto@ospedale.perugia.it (M.C.B.); daniele.rosignoli@ospedale.perugia.it (D.R.); lisa.malincarne@ospedale.perugia.it (L.M.); daniela.francisci@unipg.it (D.F.)

**Keywords:** infective endocarditis, Duke Criteria, multimodal imaging, 18 FDG-PET/CT, ESC guidelines

## Abstract

Background: The 2023 European Society of Cardiology (ESC) guidelines for the management of infective endocarditis (IE) highlighted the essential role of multimodal imaging in the diagnostic algorithm of IE and its complications. Methods: We hereby report a case series of IE in which the diagnosis was confirmed or excluded by the use of multimodal imaging during the period between January 2024 and July 2024 at the Infectious Diseases Clinic, Perugia Hospital, Italy. Results: Six patients were retrospectively included. Prosthetic valve endocarditis (PVE) was suspected in four patients and native valve endocarditis (NVE) in two cases. In patients with prosthetic valves, 18F FDG-PET/CT was performed, except in one case (P1) where cardiac CTA was performed for suspicion of perigraft aneurysm. Patients underwent transesophageal echocardiography (TOE), which was diagnostic in two cases and inconclusive in the remaining cases. In case of inconclusive TOE, the use of multimodal imaging added a major criterion and allowed us to consider (from ‘rejected’ to ‘possible’) or confirm (from ‘possible’ to ‘definite’) the diagnosis of EI based on the 2023 Duke–ESC Criteria. In one case (P6), it was possible to exclude the diagnosis. For patients with diagnostic TOE, 18F FDG-PET/CT allowed for the enhancement of diagnostic accuracy, identifying the site of valve involvement and the extension of the infection to the device (cases P3 and P5, respectively). Conclusions: In clinical practice, the use of cardiac CTA and/or 18F FDG-PET/CT based on the latest ESC guidelines demonstrated a significant impact on the diagnosis and therapeutic management of IE.

## 1. Introduction

The diagnosis of infective endocarditis (IE) is a formidable challenge due to heterogeneous clinical presentations and requires a multidisciplinary approach in both diagnosis and treatment management [1]. Epidemiology has evolved with more acute forms, different microorganisms, and an increased prevalence associated with the use of valvular prostheses and/or electronic devices [1]. The diagnosis of IE is based on clinical suspicion, combined with microbiological data and imaging evidence of endocardial lesions [1,2]. Echocardiography plays a key role as the first-line imaging modality for the diagnosis of IE and the assessment of structural and functional damage to cardiac structures [1,2]. However, echocardiography has important limitations, and advanced imaging techniques are increasingly improving the diagnostic possibilities in clinical practice [2,3]. The 2023 European Society of Cardiology (ESC) guidelines for the diagnosis and treatment of IE encourage the use of multimodal imaging techniques, for both the diagnosis of cardiac involvement, such as cardiac computed tomography angiography (CTA); fluorine 18–fludeoxyglucose (18F-FDG) positron emission tomography (PET)/CT; and/or white blood cell (WBC) single-photon emission tomography (SPECT); and the diagnosis of distant lesions, such as cerebral magnetic resonance imaging (MRI), whole-body CT, and/or PET/CT [2]. Advanced imaging techniques provide additional useful information to characterize the extent of cardiac lesions and diagnose extracardiac complications [2]. In fact, the 2023 ESC recommendations emphasize the role of 18F-FDG PET/CT and cardiac CTA as major criteria for the diagnosis of IE [2], together with microbiological findings, to improve the previous 2015 Duke–ESC Criteria [4]. Depending on the suspicion of prosthetic valve endocarditis (PVE) or native valve endocarditis (NVE), different diagnostic strategy algorithms have been proposed. Specifically, cardiac CTA is recommended for both NVE and PVE to identify valvular, paravalvular, and/or periprosthetic lesions when TOE is inconclusive or infeasible (Class I recommendation, Level of Evidence B) [2]. Additionally, 18F-FDG PET/CT is recommended in suspected IE associated with PVE for the detection of valvular lesions and confirmation of the diagnosis of IE (Class I recommendation, Level of Evidence B) [2]. It is also considered to diagnose suspected cardiovascular implanted electronic device (CIED)-related IE (Class IIb recommendation, Level of Evidence B) when echocardiography remains inconclusive [2]. In case of high clinical suspicion of PVE, WBC SPECT/CT represents an alternative nuclear imaging technique when PET/CT is not available (Class IIa recommendation, Level of Evidence C) [2]. Specifically, 99 mTechnetium-hexamethylpropyleneamine oxime (99 mTc-HMPAO)-SPECT/CT reduces the number of suspected cases of IE classified in the ‘possible IE’ category according to the modified Duke Criteria by 27% [2]. The use of an optimal imaging strategy depends on the availability and expertise of each technique [5]. However, when indicated, a multimodal imaging approach is essential in the case of suspected IE and should be strongly encouraged in the diagnostic algorithm [1,2].

The purpose of this study is to describe the cases of suspected IE in which diagnosis was confirmed or excluded by means of the use of multimodal imaging techniques in our clinical practice.

## 2. Materials and Methods

This is a retrospective case series study of consecutive patients with suspected IE, whose diagnosis was confirmed or excluded by means of the use of multimodal imaging methods, referred to the Infectious Diseases Clinic, Santa Maria della Misericordia Hospital, Italy, from January 2024 to July 2024. From the electronic medical records of the patients, demographic, clinical, imaging, microbiological, surgical, and therapeutic data were extracted. Cases of suspected IE were classified before and after the use of advanced imaging techniques, such as cardiac CTA and 18F-FDG PET/CT, as ‘definite’ IE, possible’ IE, or ‘rejected’ IE, according to the 2023 Duke–ESC Criteria [2]. In collaboration with the Department of Radiology and Nuclear Medicine, these techniques have been strongly implemented in our IE diagnostic algorithm since January 2024. Echocardiographic findings were defined as inconclusive when not specific to IE according to the main echocardiographic findings for the diagnosis and evaluation of local complications of IE of the 2023 ESC guidelines [2] and the evaluation of our Center cardiologists. According to the ESC 2023 algorithm for the diagnosis of NVE [2], cardiac CTA was performed when the echocardiography was inconclusive. Taking into account the recommended sequence of imaging techniques for PVE [2], after inconclusive echocardiography, 18F-FDG PET/CT was performed, and if the findings were diagnostic, we did not perform cardiac CTA. Cardiac CTA was performed as the first choice to diagnose paravalvular or periprosthetic complications in suspected PVE and NVE if TOE was not conclusive [2].

Personal and clinical data were processed according to the Declaration of Helsinki and the General Data Protection Regulation (679/2016).

## 3. Results

Six patients were retrospectively included, whose clinical characteristics and radiological findings are summarized in Table 1 and Table 2 and described below.

### 3.1. Case 1

Patient 1 (P1), an 88-year-old woman, was admitted to the hospital on 21 June 2024 because of persistent fever up to 40 °C started on 16 June 2024. Her medical history included ischemic heart disease (IHD), biological aortic valve prosthesis, hypertension, and diabetes mellitus type II. No known allergies were reported. An initial workup including clod cultures, autoantibodies, and rheumatoid factor were negative. In consideration of the risk of IE due to the presence of a bioprosthetic valve, the patient underwent TOE, and a not clearly defined sac was documented at the level of the aortic annulus/mitral–aortic junction. In this phase, the 2023 Duke Criteria [2] resulted in a ‘rejected’ IE, since only two minor criteria were present: fever and predisposing cardiac condition. Cardiologists considered the TOE findings worthy of further investigation with cardiac CTA, and, in this case, they did not suggest TTE. Indeed, according to the 2023 ESC guidelines [2], TOE is more sensitive and specific than TTE for the diagnosis of perivalvular complications [2]. Antibiotic therapy with ertapenem 1 g/day and daptomycin 850 mg/day was started on the day of TOE on 25 June. Cardiac CTA documented the presence of a paravalvular perigraft pseudoaneurysm. A major criterion was added, and the diagnosis of ‘possible’ blood culture-negative aortic PVE was made. Specifically, blood culture-negative infective endocarditis (BCNIE) is defined as IE in which no causative microorganism can be grown using the usual blood culture methods [2]. No infectious foci or septic emboli were documented by high-resolution chest CT, cerebral CT, and abdominal ultrasound. Nonsurgical treatment was performed according to the assessment of the Cardiac Surgery Unit, considering peri-operative risk and the potential to recover from the infection [2]. The patient completed a 6-week antibiotic therapy with the resolution of the clinical picture. Echocardiographic control demonstrated regression of paravalvular findings.

### 3.2. Case 2

Patient 2 (P2), an 84-year-old man, was admitted to the hospital on 11 April 2024 for thrombocytopenia. The patient’s medical history included IHD, transcatheter aortic valve implantation (TAVI) in January 2024, heart failure with preserved ejection fraction (HFpEF), cardiac amyloidosis, hypertension, chronic kidney disease (CKD), and idiopathic thrombocytopenia. He denied drug allergies. On 23 April, the patient presented febrile with positive blood cultures for multi-sensitive *Enterococcus faecalis*; therefore, ‘possible’ IE was suspected according to the presence of one major and two minor 2023 Duke Criteria [2]: fever up to 38 °C, predisposing heart condition, and positive blood cultures for *E. faecalis*. Moderate prosthetic aortic valve insufficiency was detected by TTE. Subsequently, the patient underwent TOE, which highlighted the hypomobility of the right cusp of the prosthetic aortic valve. On 07 May, 18F-FDG PET/CT was performed: hypermetabolism (SUV 4.2–6.2) at the level of the prosthetic aortic valve as a result of the septic process and indicative of vegetation. Due to these findings, a major criterion was added to the Duke Criteria. This changed the diagnosis from ‘possible’ to ‘definite’ IE (two major and two minor criteria). Pharmacological treatment included ceftriaxone (2 g/die) and ampicillin (3 g q12 h, due to CKD) for six weeks of treatment from the first negative blood culture. Antibiotic therapy was started on the day of the TOE on 23 April. Chest and cerebral CT and abdominal ultrasound were performed since admission to the hospital and did not reveal infectious foci or septic emboli, nor did PET/CT. The patient underwent TAVI explantation on 30 May, and biological aortic valve implantation as the follow-up TOE documented a severe periprosthetic leak. Indeed, urgent surgery is recommended for locally uncontrolled infections [2]. The subsequent TOE was negative as the bacterial cultures of the explanted TAVI material.

### 3.3. Case 3

Patient 3 (P3), an 86-year-old man, was admitted to the hospital on 4 January 2024 presenting with chest pain and a fever of up to 38.2 °C started on the same day. The patient’s medical history was significant for ascending aorta replacement, prosthetic aortic valve, IHD, hypertension, cerebral ischemic stroke (IS), and CKD. No drug allergies were reported. In order to investigate clinical manifestations on admission, a TTE was performed and a perivalvular jet around the aortic prosthesis was detected. Subsequently, TOE revealed a vegetation (3 mm × 4 mm) of the left ventricular outflow tract (LVOT) and a pseudoaneurysm of the mitral–aortic junction. Considering the 2023 Duke Criteria [2], a diagnosis of ‘possible’ IE was made based on the presence of one major and two minor criteria: vegetation detected by TOE, fever up to 38 °C, and predisposing cardiac disease. All blood cultures were sterile, as in BCNIE. Notably, 18F-FDG PET/CT showed hypermetabolism of the aortic prosthesis, which was indicative of vegetation, and further characterized the site of the valvular lesion. The study also excluded the presence of vegetation on the ascending aorta prosthesis. According to the Duke Criteria [2], the diagnosis of ‘possible’ IE remained, but the site of vegetation could be better identified. Abdomen CT, cerebral MRI, and chest X-ray, as well as PET/CT, excluded the presence of infectious foci or septic emboli. A 6-week course of daptomycin (850 mg/day) and ceftriaxone (2 g/die) was administered after the TOE findings. Only one week of gentamicin 3 mg/kg every 48 h was administered due to the rapid worsening of renal failure. The patient was not a candidate for valvular surgery because of the high operative risk. The follow-up echocardiography evaluation showed an improvement in the pathological findings.

### 3.4. Case 4

Patient 4 (P4), an 85-year-old woman, was admitted to the hospital on 1 July 2024 with a diagnosis of congestive heart failure. The patient had a cardiovascular history of the prosthetic aortic valve, reduced ejection fraction heart failure (HFrEF), hypertension, permanent atrial fibrillation (AF), and bilateral carotid atheromasia. On 15 July, she developed a fever with chills, and blood cultures were taken and were positive for methicillin-resistant Staphylococcus epidermidis (MRSE). TTE was negative for suspected lesions consistent with IE. On 25 July, TOE was performed, and the absence of images suggestive of IE was confirmed. According to the 2023 Duke Criteria [2], ‘possible’ IE was considered, with three minor criteria: fever (>38 °C), cardiac predisposition, and positive blood cultures for MRSE. Additionally, 18F-FDG PET/CT was performed on 3 August and documented the presence of a small hypermetabolic spot projecting at the level of the anterior prosthetic aortic valve, suggestive of an infectious process at the periprosthetic aortic valve site. Due to this finding, a major criterion was added, which changed the diagnosis to ‘definite’ IE. Abdominal and chest CT, as well as PET/CT, excluded the presence of infectious foci or septic emboli. Empiric therapy with daptomycin (700 mg/day) and ceftriaxone (2 g/day) was started on 15 July (before TOE) and was changed to daptomycin alone for 6 weeks after MRSE isolation. A follow-up 18F-FDG PET/CT was performed on 12 September and documented a reduction in extent and gradient uptake. According to the Cardiac Surgery Unit, TAVI was performed on 10 August, whereas bacterial cultures of the explanted PMK were not performed.

### 3.5. Case 5

Patient 5 (P5), a 54-year-old man, was admitted to the hospital on 10 February 2024, with edema, erythema, and pain in the lower limbs. The patient’s medical history showed severe mitral valvular disease repaired by annuloplasty, sick sinus syndrome (SSS) treated with pacemaker (PMK) implantation in 2017, and atrial flutter; he also denied drug allergies. Considering the clinical suspicion of acute bacterial skin and skin structure infection (ABSSSI), multiple blood cultures were taken, and empiric therapy with ceftriaxone (2 g/die) was started. Subsequently, blood cultures collected on 10 February resulted positive for *Salmonella* Group C of unknown origin. Furthermore, stool bacterial cultures, colonoscopy, and esophagogastroduodenoscopy were negative. Considering the atypical presentation and the detected pathogen, IE was investigated. Both TTE and TOE showed the presence of mitral valve vegetation (multiple fluctuating filiform formations, the largest of which fluctuated from the anterior valve leaflet). Antibiotic therapy with ceftriaxone (2gr q12) was continued, and ciprofloxacin (400 mg q12) was added. Whole-body CT revealed splenic and renal septic emboli, adding one minor criterion. The Duke Criteria [2] revealed a ‘definite’ IE (one major and three minor criteria): vegetation on mitral valve, heart predisposition, septic emboli, and positive blood cultures for Salmonella group C (nontypical microorganism). Additionally, 18F-FDG PET/CT was performed, and uptake was detected at the mitral valve level and the pacemaker lead. These findings did not change the diagnosis of ‘possible’ IE. However, they documented the extent of the infection to the device. After consulting the cardiac surgeons and assessing the risks involved, it was decided to proceed with PMK and lead replacement on 12 March. The definitive PMK reimplantation occurred on 12 April. The pacemaker lead cultures were performed but demonstrated no growth at 5 days. Following the procedure, suppressive antibiotic therapy (SAT) was administered with trimetoprim/sulfametoxazolo (TMP/SMX) after 6 weeks of ceftriaxone and ciprofloxacin, which was started after positive TTE. Follow-up 18F-FDG PET/CT and TOE were performed on 25 and 27 March, respectively, and the persistence of mitral valve vegetations was documented, and SAT was continued.

### 3.6. Case 6

Patient 6 (P6), a 75-year-old man, was admitted to the hospital on 13 May 2024 with a diagnosis of COVID-19 pneumonia. The patient’s medical history included IS, hypertensive heart disease, high-grade atrioventricular block requiring PMK implantation, abdominal aortic ectasia, and dyslipidemia; he also denied drug allergies. Because he was febrile on 27 May, multiple blood cultures were taken, which tested positive for *Candida parapsilosis*. The diagnostic algorithm was applied to rule out cardiac involvement. The TEE excluded vegetation of the valve and the pacemaker lead, and the TOE documented a hyperechogenic, oval, fluctuating formation in the atrial cavity close to the PMK. These findings raised the suspicion of ‘possible’ IE due to the presence of two minor and one major 2023 Duke Criteria [2]. Specifically, cardiac predisposition, positive blood cultures for *C. parapsilosis*, and suspected vegetation in the atrial cavity were considered major criteria. On 17 July, 18F-FDG PET/CT showed no uptake at the cardiac/valvular and pacemaker level, which ruled out the suspicion of IE according to the 2023 Duke Criteria [2]. Whole-body CT and cerebral MRI excluded infectious foci or septic emboli, and fundoscopic examination was negative. In this case, the predisposing factors for candidemia were prior antibiotic exposure and high steroid administration due to COVID-19 pneumonia. Treatment for *C. parapsilosis* was administered with caspofungin (50 mg/day) at the same time as when blood cultures were positive, before echocardiographic findings. Follow-up TOE was negative.

## 4. Discussion

Six patients were included in this study, four of whom had suspected PVE, and two had suspected NVE, with suspected CIED involvement in two cases (P5 and P6) and TAVI in one case (P2). In terms of predisposing factors, TAVI was included as a high-risk condition for IE, and CIEDs were considered intermediate risk, as they account for a significant proportion of IE cases with a high risk of morbidity and mortality according to the 2023 ESC guidelines [2].

All patients underwent TTE and/or TOE, which were diagnostic in two cases (P3 and P5) and inconclusive in the remaining cases. Specifically, in one case (P5), both TTE and TOE documented vegetation on the native mitral valve, whereas in the other case (P3), TEE was inconclusive, and TOE was diagnostic for vegetation in the LVOT in the presence of an aortic valve prosthesis. The transthoracic modality is the recommended first-line approach, but it may only be sufficient for the diagnosis of perivalvular complications, small vegetation, PVE, and vegetation associated with CIEDs [2,3,5]. Transesophageal modality is superior to TTE in these aspects and is recommended in patients with inconclusive or negative TTE and high suspicion of IE, as well as in patients with positive TTE, to detect local complications [2,3,5]. Both TTE and TOE can document the location and size of the vegetation, assess the paravalvular extension of the infection, and define the hemodynamic effects of valvular or device infection [2,3,5].

In our study, the echocardiographic findings were inconclusive and/or negative in four cases, particularly in the case of suspected PVE. In this scenario, cardiac CTA was performed in patients with suspicion of NVE, while 18F-FDG-PET/CT was performed in cases of prosthetic valve and/or device involvement, in accordance with 2023 ESC recommendations [2]. In one case (P1) of suspected PVE, cardiac CTA was performed because of suspicion of a perigraft aneurysm. For the assessment of paravalvular complications of IE, such as abscesses and pseudoaneurysms, cardiac CTA is more accurate than TOE [2,3,6,7]. In general, the sensitivity of 18F-FDG PET/CT is much lower in NVE than in PVE or CIED-related IE [2,3,4,5,6,7,8]. These findings depend on the higher degree of fibrosis in NVE compared to PVE; the impact of the persistent inflammatory environment due to biofilm on prosthetic valves; and the thicker valvular apparatus associated with prosthetic valves compared to the thin and highly mobile leaflets of native valves [9,10]. In PVE and/or CIED-related IE, 18F-FDG PET/CT is helpful in demonstrating periprosthetic hypermetabolism when echocardiography is inconclusive [2,11]. Pathological findings suggestive of infection refer to patterns of high-intensity focal or heterogeneous valvular/prosthetic or perivalvular/periprosthetic uptake [2,11].

In our study, 18F-FDG PET/CT and cardiac CTA allowed us to optimize diagnostic and therapeutic management by improving the diagnostic definition of cases of suspected IE, according to the 2023 Duke Criteria [2]. Specifically, in the case of inconclusive TOE, the use of advanced imaging modalities added a major criterion and allowed us to consider (from ‘rejected’ to ‘possible’) in P1 or confirm (from ‘possible’ to ‘definite’) the diagnosis of IE in cases P2 and P4. In one case (P6), the diagnosis was excluded (from ‘possible’ to ‘rejected’) with implications for therapeutic management. As all of these cases referred to PVE or CIED involvement, 18F-FDG PET/CT had a discriminatory role in the modification of the diagnosis. Furthermore, in one case (P4), it has also helped follow-up management, as both TTE and TOE were negative.

In patients with diagnostic TOE and suspected or definite PVE or CIED involvement, 18F-FDG PET/CT allowed for an increase in diagnostic accuracy, identifying the site of prosthetic valve involvement and the extension of infection to the device in cases P3 and P5, respectively.

In the reclassification of IE from ‘possible’ to ‘definite’, 18F-FDG PET/CT contributed up to 90% [3]. In a Swiss multicenter cohort, the accuracy of the 2023 Duke–ESC Criteria was up to 90%, which was attributed to the inclusion of factors such as CIED and TAVI as predisposing factors and the detection of abnormal metabolic activity on 18F-FDG PET/CT [12]. Furthermore, 18F-FDG PET/CT has improved diagnostic accuracy, particularly in detecting extracardiac foci [13]. It has also played an important role in the prediction of prognosis and the risk of embolic events in case of increased FDG uptake in the vicinity of the tissues and elevated C-reactive protein (CRP) levels [13].

In this study, despite the routine use of whole-body multimodal imaging to search for distant lesions, only one patient (P5) met the minor criteria for embolic vascular dissemination or immunological phenomena, adding one minor criterion and confirming the diagnosis of IE thanks to whole-body CT. In this setting, in both symptomatic (Class I recommendation, Level of Evidence B) and asymptomatic (Class IIb recommendation, Level of Evidence B) patients with PVE or NVE, the use of multimodal imaging techniques had the role of adding a minor diagnostic criterion [2].

Caporali et al. analyzed several evidence-based studies on the clinical role of multimodal imaging and remarked that the addition of 18F-FDG PET/CT to the standard workup of IE detected 10–50% of potential infectious foci and improved the management of one-third of patients [14]. Despite the numerous advantages of multimodal imaging, it is important to highlight its potential pitfalls such as inconsistent and difficult-to-interpret imaging results. Specifically, cardiac CTA is affected by low sensitivity for small vegetation and perforations compared to TEE, increased radiation exposure, contrast administration, and longer scan time compared to echocardiography [2,15]. 18F-FDG PET/CT demonstrated limited specificity, as uptake can also occur in non-infective conditions; limited availability; higher cost; the need for expertise in performing the exam and interpreting the results; and limited value in assessing cerebral complications [2,14,15]. Similarly, WBC-SPECT is also hampered by limited availability, higher cost compared to conventional imaging modalities, and lower image resolution and overall sensitivity [2,15]. Regarding future prospects, machine learning and artificial intelligence (AI) are emerging important tools for the diagnosis of IE [14,15]. Specifically, the role of both of these technologies is to improve the diagnostic performance of the Duke Criteria [2] and optimize the interpretation of advanced imaging modalities findings [15]. Furthermore, novel PET tracers [14] and other multimodality approaches such as PET/MRI and leucocyte scintigraphy/MRI will be explored in future studies on PVE and (CIED)-related IE [15].

The strength of this case series includes its real-world setting which may be helpful in understanding the 2023 ESC guidelines [2] and implementing diagnostic algorithms in clinical practice; however, it has several limitations. The retrospective design and small sample size limited the full comprehension of the impact of advanced imaging techniques in real-world scenarios. Furthermore, we did not provide the iconography of the multimodal imaging findings detected for each case. Further research is expected to support the currently available results in IE imaging diagnosis, which are not yet fully investigated: this could help select the most suitable patients for each advanced imaging technique.

## 5. Conclusions

This case series documented the positive impact of cardiac CTA and/or 18F-FDG PET/CT based on the latest ESC guidelines for the diagnosis and therapeutic management of PVE, NVE, and TAVI or CIED involvement. Specifically, in cases of nonconclusive TOE, it allowed the diagnosis to be confirmed or excluded. In cases of diagnostic TOE, it characterized the extension and location of the endocarditis lesions. According to ESC 2023 diagnostic algorithms [2], it is fundamental to know the strengths and limitations of each of these modalities to clearly enhance the multimodal imaging approach in clinical practice and increase diagnostic accuracy.

## Figures and Tables

**Table 1:** Microbiological and radiological findings of the study population.

	Blood Cultures	TTE	TOE	Cardiac CTA	18F FDG-PET/CT
P1 F88	Negative	Not performed	Inconclusive (paravalvular aortic sac)	Paravalvular perigraft pseudo-aneurysm	Not performed
P2 M84	*Enterococcus* *faecalis*	Inconclusive (prosthetic aortic valve moderate insufficiency)	Inconclusive (hypomobility of the right aortic cusp)	Not performed	Hypermetabolism of the aortic right cusp indicative of vegetation
P3 M86	Negative	Inconclusive (perivalvular jet around the aortic prosthesis)	Fluctuating vegetation in the LVOT	Not performed	Hypermetabolism of the aortic valve prosthesis indicative of vegetation
P4 F85	Methicillin-resistant *Staphylococcus epidermidis*	Negative	Negative	Not performed	Hypermetabolism of the aortic valve prosthesis indicative of vegetation
P5 M54	*Salmonella* Group C	Mitral valve vegetations	Mitral valve vegetations	Not performed	Hypermetabolism of the mitral valve and PMK lead indicative of vegetation
P6 M76	*Candida* *parapsilosis*	Negative	Inconclusive (aspecific formation in atrial cavity near the PMK)	Not performed	Negative

P: patient; F: female; M: male; LVOT: left ventricular outflow tract; TTE: transthoracic echocardiography; TOE: transesophageal echocardiography; cardiac CTA: cardiac computed tomography angiography; 18F-FDG PET/CT: fluorine 18–fludeoxyglucose (18F-FDG) positron emission tomography (PET)/CT; PMK: pacemaker.

**Table 2:** The 2023 Duke–ESC Criteria [2] classification and clinical outcome of the study population.

	2023 Duke–ESC Criteria PRE/POST Multimodal Imaging	Diagnosis	Therapy Surgery	Outcome
P1 F88	PRE: Rejected 2 m POST: Possible 1Ma + 2 m	PVE possible aortic biological valve	Daptomycin + ertapenem 6 wk No surgery	Recovery
P2 M84	PRE: Possible 1Ma + 2 m POST: Definite 2Ma + 2 m	PVE definite TAVI	Ampicillin + ceftriaxone 6 wk, Biological aortic valve implantation	Recovery
P3 M86	PRE: Possible 1Ma + 2 m POST: Possible 1Ma + 2 m	PVE possible aortic mechanical valve	Ceftriaxone + daptomycin 6 wk No surgery	Recovery
P4 F85	PRE: Possible 3 m POST: Definite 1Ma + 3 m	PVE definite aortic mechanical valve	Daptomycin 6 wk TAVI	Recovery
P5 M54	PRE: Definite 1Ma + 3 m POST: Definite 1Ma + 3 m	NVE definite mitralic valve and PMK lead	Ceftriaxone+ ciprofloxacin 6 wk PMK replacement	SAT TMP/SMX
P6 M76	PRE: Possible 1Ma + 2 m POST: Rejected 2 m	NVE rejected PMK not infected	Caspofungin LD 70 mg, then 50 mg/die 2 wk No surgery	Recovery

P: patient; F: female; M: male; Ma: major criterion; m: minor criterion; wk: weeks; LD: loading dose; TMP/SMX: trimetoprim/sulfametoxazolo; SAT: suppressive antibiotic therapy; TAVI: transcatheter aortic valve implantation; PVE: prosthetic valve endocarditis; NVE: native valve endocarditis; PMK: pacemaker.

## Data Availability

The data that supports the findings of this study are available from the corresponding author, S.T., upon reasonable request.
